# Use of Human Albumin Administration for the Prevention and Treatment of Hyponatremia in Patients with Liver Cirrhosis: A Systematic Review and Meta-Analysis

**DOI:** 10.3390/jcm11195928

**Published:** 2022-10-08

**Authors:** Zhaohui Bai, Le Wang, Hanyang Lin, Frank Tacke, Gang Cheng, Xingshun Qi

**Affiliations:** 1 NMPA Key Laboratory for Research and Evaluation of Drug Regulatory Technology, Shenyang Pharmaceutical University, Shenyang 110016, China; 2 Liver Cirrhosis Study Group, Department of Gastroenterology, General Hospital of Northern Theater Command, Shenyang 110840, China; 3 Department of Hepatology & Gastroenterology, Charité University Medical Center, 10117 Berlin, Germany

**Keywords:** liver cirrhosis, hyponatremia, sodium, serum albumin, human

## Abstract

Background. Hyponatremia is a common complication of liver cirrhosis and aggravates patients’ outcomes. It may be corrected by human albumin (HA) infusion. Herein, we have conducted a systematic review and meta-analysis to evaluate the efficacy of intravenous HA administration for the prevention and treatment of hyponatremia in liver cirrhosis. Methods. Literature was searched in the PubMed, EMBASE, and Cochrane Library databases. If possible, a meta-analysis would be conducted. Incidence of hyponatremia, rate of resolution of hyponatremia, and serum sodium level were compared between cirrhotic patients who received and did not receive HA infusion. Odds ratios (ORs) or mean differences (MDs) with 95% confidence intervals (CIs) were calculated. The quality of evidence was assessed by the Grading of Recommendations Assessment, Development, and Evaluation (GRADE) system. Results. Initially, 3231 papers were identified. Among them, 30 studies, including 25 randomized controlled trials (RCTs) and 5 cohort studies, were eligible. Among cirrhotic patients without hyponatremia, the HA infusion group had significantly lower incidence of hyponatremia (OR = 0.55, 95%CI = 0.38–0.80, *p* = 0.001) and higher serum sodium level (MD = 0.95, 95%CI = 0.47–1.43, *p* = 0.0001) as compared to the control group. Among cirrhotic patients with hyponatremia, the HA infusion group had a significantly higher rate of resolution of hyponatremia (OR = 1.50, 95%CI = 1.17–1.92, *p* = 0.001) as compared to the control group. Generally, the quality of available evidence is low. Conclusions. Based on the current evidence, HA may be considered for preventing the development of hyponatremia in liver cirrhosis, especially in those undergoing LVP, and treating hyponatremia. Well-designed studies are required to clarify the effects of HA infusion on hyponatremia in liver cirrhosis.

## 1. Introduction

**Figure 1:**
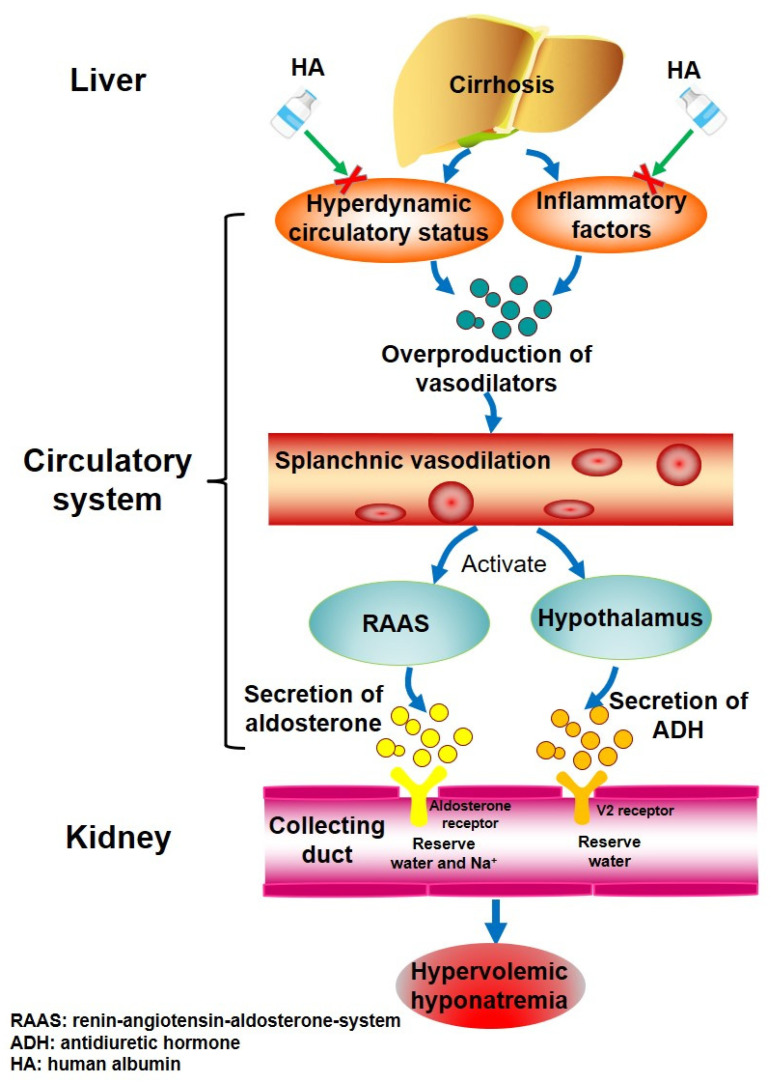
Potential mechanisms of human albumin infusion on hyponatremia.

Hyponatremia, a common complication of liver cirrhosis [1], is divided into mild (serum sodium level 135–130 mmol/L), moderate (130–125 mmol/L), and severe (<125 mmol/L) [2]. The prevalence of serum sodium levels of <135 mmol/L, <130 mmol/L, <125 mmol/L, and <120 mmol/L is 49.4%, 21.6%, 5.7%, and 1.2% in total cirrhotic patients, respectively [3]. Mild hyponatremia is often asymptomatic; by comparison, moderate and severe hyponatremia can cause nausea, cognitive impairment, headache, and even coma [4]. Hyponatremia is classified as hypovolemic, euvolemic, and hypervolemic according to the volume status on the clinical examinations [1]. In patients with liver cirrhosis, about 90% of hyponatremia is hypervolemic, which is primarily due to increased extracellular fluid volume (Figure 1), and the remaining 10% is hypovolemic or euvolemic, which is frequently caused by over-diuresis [5]. Hyponatremia is significantly associated with worse outcomes in liver cirrhosis [6,7,8]. More importantly, serum sodium level has been incorporated into the model for end-stage liver disease (MELD) score, which is defined as the MELD-Na score, to determine the priority of liver transplantation [9].

Currently, water restriction and isotonic saline are the mainstay treatment options for hyponatremia, but their efficacy is limited [10]. Additionally, discontinuation of diuretics [11], correction of hypokalemia [12,13], and use of vasopressin receptor antagonists [14,15] have been attempted to manage mild and moderate hyponatremia, but their efficacy and safety remain to be further validated. Hypertonic saline is only reserved for severe symptomatic hyponatremia, such as seizure, cardiopulmonary distress, and coma [16].

The use of human albumin (HA) in the management of liver cirrhosis with hyponatremia remains controversial among the current practice guidelines. Japanese and Italian practice guidelines suggest that HA should be used to prevent and/or treat hyponatremia in liver cirrhosis [17,18], but the American Association for the Study of Liver Diseases (AASLD), European Association for the Study of the Liver (EASL), and Chinese guidelines have not recommended the use of HA in such cases yet [19,20,21]. The heterogeneity in the guideline recommendations may be attributed to the scarcity of high-quality evidence.

To the best of our knowledge, only several previous meta-analyses partly evaluated the role of HA infusion in the prevention of hyponatremia in liver cirrhosis after large volume paracentesis (LVP) [22,23,24,25,26,27], but the conclusions were heterogeneous among them. Additionally, the preventive and therapeutic effects of HA infusion on hyponatremia in general liver cirrhosis have not been systematically evaluated. Herein, we attempted to collect all existing evidence regarding this topic and combine the relevant data to further address this issue.

## 2. Methods

### 2.1. Registration

The current study was registered in the PROSPERO. The registration number is CRD42021233576.

### 2.2. Literature Search

We searched 3 major electronic databases (i.e., PubMed, EMBASE, and Cochrane Library). The last search date was 13 September 2022. Search items were as follows: ((colloid [All Fields]) OR (albumin [All Fields]) OR (HSA [All Fields])) AND ((cirrhosis [All Fields]) OR (cirrhotic [All Fields])) AND ((hyponatremia [All Fields]) OR (hyponatraemia [All Fields]) OR (sodium [All Fields]) OR (Na [All Fields])).

### 2.3. Study Selection Criteria

Two researchers (Dr. Bai and Dr. Wang) individually selected eligible studies by screening the title, abstract, and full text. Studies would be eligible for inclusion if they explored the effect of HA on the prevention or treatment of hyponatremia in cirrhotic patients. Exclusion criteria were as follows: (1) duplicates; (2) guidelines, reviews, or meta-analyses; (3) case reports, comments, or letters; (4) experimental or animal studies; (5) studies unrelated to liver cirrhosis; (6) studies unrelated to HA; (7) studies unrelated to hyponatremia; (8) the sample size was less than 10.

### 2.4. Definitions and Outcomes

Definitions of hyponatremia in liver cirrhosis are in accordance with those of each included study. The primary outcome is the incidence of hyponatremia after treatment. The secondary outcome is serum sodium level after treatment.

### 2.5. Data Extraction

Data were extracted by 2 researchers (Dr. Bai and Dr. Wang) from each study, mainly including first author, publication year, country, characteristics of patients, sample size, etiology of liver cirrhosis, the definition of hyponatremia, dosage of HA infused, the total number of patients, number of patients with hyponatremia, and serum sodium level after treatment. Disagreement was resolved by discussion among researchers.

### 2.6. Study Quality Assessment

For randomized controlled trials (RCTs), the Cochrane Risk of Bias tool was used to assess the risk of bias, which includes random sequence generation, allocation concealment, blinding of participants and personnel, blinding of outcome assessment, incomplete outcome data, selective reporting, and other bias. For cohort studies, the Newcastle-Ottawa Scale (NOS) was used to assess the study quality, which includes 3 parts (i.e., selection, comparability, and outcomes) and 8 indicators. High-quality cohort studies are defined if 5 or more points are given.

### 2.7. Statistical Analysis

Meta-analysis was performed by the Review Manager software (Version 5.3, The Nordic Cochrane Centre, The Cochrane Collaboration, Copenhagen, Denmark) and Stata SE (Version 12.0, Stata Corp, College Station, TX, USA). Dichotomous outcomes were expressed as odds ratios (ORs) with 95% confidence intervals (CIs), and continuous outcomes were expressed as mean differences (MDs) with 95% CIs. A random-effects model was employed. A *p*-value < 0.05 was considered statistically significant. Cochrane Q test and I² statistics were employed to assess the heterogeneity, and a *p*-value < 0.1 or I² > 50% was considered a statistically significant heterogeneity. Sensitivity analyses, meta-regression analyses, and subgroup analyses were used to explore the source of heterogeneity. Meta-regression analyses were performed by 5 covariates, which included publication year (before and after 2000), region (Asia, Europe, America, and Africa), sample size (> and <100), type of control group, and LVP or not. Subgroup analyses were also conducted in terms of the above-mentioned variables. When there were ≥10 studies included in a meta-analysis, the publication bias was assessed by a visual assessment of funnel plot asymmetry [28,29,30]. The Grading of Recommendations Assessment, Development and Evaluation (GRADE) system [31] was employed to assess the quality of evidence for the meta-analysis.

## 3. Results

### 3.1. Study Selection

**Figure 2:**
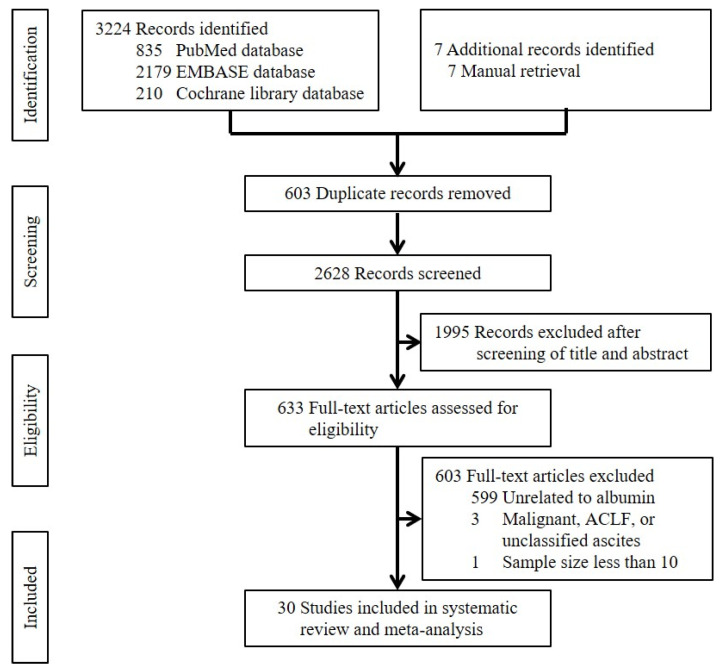
Flow chart of study selection.

Overall, we identified 3231 papers from the PubMed, Embase, and Cochrane Library databases and manual retrieval. Thirty-four studies were potentially eligible. Notably, four studies were further excluded, because they explored malignant ascites, acute-on-chronic liver failure with ascites, and unclassified ascites [32,33,34], or the sample size was less than 10 [35]. Finally, 30 studies were included [36,37,38,39,40,41,42,43,44,45,46,47,48,49,50,51,52,53,54,55,56,57,58,59,60,61,62,63,64,65] (Figure 2).

### 3.2. Study Characteristics

The characteristics of included studies are listed in Table 1 and Table S1. Twenty-nine studies were published as full texts and one as abstract. According to the countries where studies were conducted, five studies were conducted in Spain [36,37,42,48,61], three in the USA [55,59,60], three in Mexico [40,41,46], three in France [43,47,49], three in Egypt [54,56,62], three in Italy [38,44,65], three in the UK [51,63,64], two in India [50,53], two in Germany [45,52], one in Argentina [39], one in Pakistan [57], and one in Iran [58]. The sample size ranged from 16 to 1126. The publication year ranged from 1988 to 2022.

### 3.3. Study Quality

Seventeen studies had low risk in random sequence generation [36,37,40,42,43,44,46,48,49,52,53,54,55,56,58,61,62], nineteen had low risk in allocation concealment [36,37,38,39,40,42,43,44,46,47,48,49,52,53,54,55,56,58,61], three had low risk in blinding of participants and personnel [53,55,61], three had low risk in blinding of outcome assessment [53,55,61], twenty-two had low risk in incomplete outcome data [36,37,38,39,40,42,43,44,46,47,48,49,50,53,54,55,56,57,58,61,62,64], twenty-three had low risk in selective reporting [36,37,38,39,40,42,43,44,46,47,48,49,50,52,53,54,55,56,57,58,61,62,64], and nine had low risk in other bias [40,42,44,46,47,49,50,61,62] (Figure S1). Among the five cohort studies, all were of high quality [45,59,60,63,65] (Table S2).

### 3.4. HA for the Prevention of Hyponatremia

#### 3.4.1. Incidence of Hyponatremia

**Figure 3:**
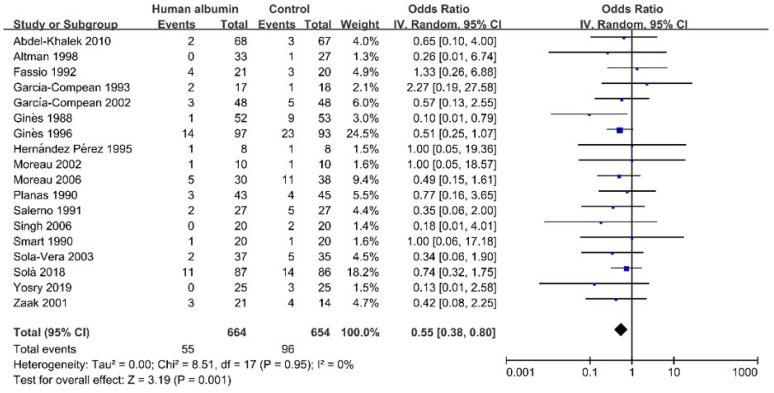
Forrest plots showing the effect of human albumin infusion on the development of hyponatremia in liver cirrhosis without hyponatremia.

Eighteen studies, including 1318 cirrhotic patients, provided data regarding the effect of HA on the development of hyponatremia [36,37,38,39,40,41,42,43,45,46,47,48,49,50,54,61,62,64]. Meta-analysis showed that the incidence of hyponatremia was significantly lower in HA infusion groups than in control groups (OR = 0.55, 95%CI = 0.38–0.80, *p* = 0.001) (Figure 3). Publication bias was not statistically significant (Figure S2A). The heterogeneity was not statistically significant (I^2^ = 0%, *p* = 0.95) (Figure 3). Thus, sensitivity, meta-regression, and subgroup analyses were not performed.

#### 3.4.2. Serum Sodium Level

**Figure 4:**
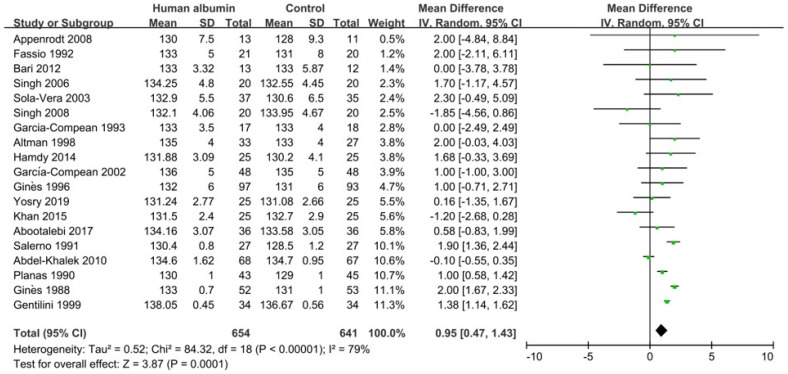
Forrest plots showing the effect of human albumin infusion on serum sodium level in liver cirrhosis without hyponatremia.

Nineteen RCTs, including 1295 cirrhotic patients, provided data regarding the effect of HA infusion on serum sodium levels [36,37,38,39,40,42,43,44,46,48,50,52,53,54,55,56,57,58,62]. Meta-analysis showed that serum sodium level was significantly higher in HA infusion groups than control groups (MD = 0.95, 95%CI = 0.47–1.43, *p* = 0.0001) (Figure 4). Publication bias was statistically significant (Figure S2B). The heterogeneity was statistically significant (I^2^ = 79%, *p* < 0.00001) (Figure 4). Sensitivity analysis did not find the source of heterogeneity (Figure S3). Meta-regression analysis found that the source of heterogeneity might be the target population (Table S3). Subgroup analyses demonstrated that the heterogeneity might be related to the type of control group because the heterogeneity was not statistically significant in the studies where dextran (I^2^ = 0%, *p* = 0.97) and midodrine (I^2^ = 33%, *p* = 0.21) were used in the control group (Table S4).

### 3.5. HA for the Treatment of Hyponatremia

#### 3.5.1. Resolution of Hyponatremia

**Figure 5:**
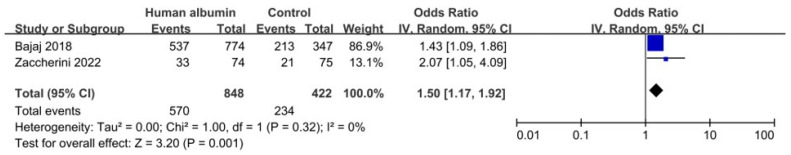
Forrest plots showing the effect of human albumin infusion on the resolution of hyponatremia in liver cirrhosis with hyponatremia.

Two studies, including 1270 cirrhotic patients, provided data regarding the effect of HA on the resolution of hyponatremia [60,65]. Meta-analysis showed that the resolution of hyponatremia was significantly more common in the HA infusion group than in the control group (OR = 1.50, 95%CI = 1.17–1.92, *p* = 0.001). Publication bias could not be evaluated, because the number of included studies was <10 in this meta-analysis. Among them, the heterogeneity was not statistically significant (I^2^ = 0%, *p* = 0.32) (Figure 5). Thus, sensitivity, meta-regression, and subgroup analyses were not performed.

#### 3.5.2. Serum Sodium Level

Three studies reported a change in serum sodium level after HA infusion in patients with hyponatremia, and all of them demonstrated significantly increased serum sodium levels after HA infusion in patients with hyponatremia. However, their data expression was so heterogeneous that a meta-analysis could not be performed. In detail, an RCT by Jalan et al. [51] reported that the mean serum sodium level after HA infusion was increased from 124 ± 2 mmol/L to 133 ± 6 mmol/L in 12 patients with serum sodium levels < 130 mmol/L; a retrospective cohort study by Shen et al. [59] reported that the mean increase in serum sodium level in HA infusion group was 5.043 ± 19.0 mmol/L in 55 patients with serum sodium level < 130 mmol/L; and secondary analysis of data from an RCT by China et al. [63] reported that the mean increase in serum sodium level when HA infusion group was compared with control group was 1.77 mmol/L (95%CI = 1.04–2.51) in 103 patients with serum sodium level < 130 mmol/L.

### 3.6. Quality of Evidence

Based on the GRADE summaries, the quality of evidence is low or very low (Table S5).

## 4. Discussion

The current systematic review and meta-analysis of 30 studies involving 3298 cirrhotic patients comprehensively explored the effect of HA on the prevention and treatment of hyponatremia. We found that HA might be considered for preventing the development of hyponatremia in liver cirrhosis, especially in those undergoing LVP, and treating hyponatremia. However, the evidence is of low quality and insufficient.

To our knowledge, six previous meta-analyses by Bernardi [22], Kwok [23], Kütting [24], Simonetti [25], Zheng [26], and Shrestha [27], primarily evaluated the efficacy of HA infusion for the prevention of post-paracentesis circulatory dysfunction in cirrhotic patients undergoing LVP, also reported the relevant data regarding its impact on the prevention of hyponatremia after LVP. By comparison, the current meta-analysis has several strengths. First, six previous meta-analyses just included cirrhotic patients undergoing LVP [22,23,24,25,26,27], but the current meta-analysis included general cirrhotic patients. Second, six previous meta-analyses just explored the prevention of hyponatremia after LVP [22,23,24,25,26,27]; by comparison, the current meta-analysis not only explored the prevention of hyponatremia, but also the treatment of hyponatremia. Third, six previous meta-analyses just pooled the incidence of hyponatremia after LVP [22,23,24,25,26,27]; by comparison, the current meta-analysis not only pooled the incidence and improvement rate of hyponatremia but also pooled the serum sodium level after treatment. Fourth, Bernardi’s [22], Kwok’s [23], Kütting’s [24], Zheng’s [26], Simonetti’s [25] and Shrestha’s [27] meta-analyses included 13, 7, 14, 17, 11, and 13 studies, respectively; by comparison, the current meta-analysis included 25 studies regarding the prevention of hyponatremia. Finally, Bernardi’s [22], Kwok’s [23], Kütting’s [24], Zheng’s [26], and Shrestha’s [27] meta-analyses did not assess the quality of evidence, but the current meta-analysis and Simonetti’s [25] meta-analysis assessed it based on the GRADE system.

HA has been widely used for various complications of decompensated cirrhosis [66], including spontaneous bacterial peritonitis [67,68], hepatorenal syndrome [69], ascites [70], and hepatic encephalopathy [71]. However, the evidence supporting its use for hyponatremia is very limited. The current meta-analysis showed that HA might be advantageous for hyponatremia in liver cirrhosis. The benefits of HA can be explained by the pathological mechanism of hyponatremia in liver cirrhosis and the physiological function of HA (Figure 1).

The pathogenesis of hyponatremia in liver cirrhosis is multifactorial. Increased intrahepatic vascular resistance leads to the development of portal hypertension in advanced liver cirrhosis, which can induce hyperdynamic circulatory status [72,73]. Additionally, inflammatory factors are significantly increased in decompensated cirrhotic patients [74]. Both of them can lead to the overproduction of vasodilators, which mainly include nitric oxide, substance P, platelet-activating factor, and prostacyclin [75]. Splanchnic vasodilation will cause hypovolemia in the peripheral circulatory system, and then activate the renin-angiotensin-aldosterone-system and the secretion of antidiuretic hormone [4,16]. Aldosterone can activate the mineralocorticoid receptor on the distal convoluted tubule and collecting duct, and then reserve water and sodium [76]. Antidiuretic hormone can activate the V2 receptor on the renal collecting duct, and then reserve a large volume of water and increase urinary sodium excretion [77,78], subsequently developing hypervolemic hyponatremia.

HA is responsible for maintaining colloid osmotic pressure and influencing inflammatory pathways [79,80,81,82]. Therefore, it may act on the upstream pathogenesis of hyponatremia by improving the hyperdynamic circulatory status and clearing inflammatory factors [83]. By comparison, dextran, hydroxyethyl starch, hemaccel, midodrine, and terlipressin could improve hypovolemia and/or hyperdynamic circulatory status, but not clear inflammatory factors.

It should be acknowledged that the therapeutic value of HA for hyponatremia in liver cirrhosis is evaluated based on only one small RCT published as an abstract [51] and four cohort studies [59,60,63,65]. Additionally, the heterogeneity in study design is obvious among them. First, in Shen’s [59], China’s [63], and Zaccherini’s [65] studies, HA was selectively infused in the control group; by comparison, in Bajaj’s [60] study, no HA infusion was given in the control group. Second, HA was infused at a total dosage of 225 g in Bajaj’s [60] study, a total dosage of 239.4 g in China’s [63] study, and a dosage of 40 g twice weekly for 2 weeks, and then 40 g weekly in Zaccherini’s [65] study, but the dosage of HA infused was unclear in Shen’s [59] study. Third, baseline serum sodium level was 126.1 ± 4 mmol/L and 128.66 ± 4.69 mmol/L in Shen’s [59] and Bajaj’s [60] studies, respectively. By comparison, 78% of patients had mild hyponatremia with a serum sodium level of 130–135 mmol/L in Zaccherini’s [65] study, and the severity of hyponatremia was unclear in China’s [63] study. Fourth, the resolution of hyponatremia was evaluated in Bajaj’s [60] and Zaccherini’s [65] studies; by comparison, the change in serum sodium level was evaluated in Shen’s [59] and China’s [63] studies.

A major limitation of the current systematic review and meta-analysis is that most included studies focused on the prevention of hyponatremia in liver cirrhosis with ascites undergoing LVP. Notably, hyponatremia is also an important risk factor for ascites without LVP [84,85,86], hepatic encephalopathy [87,88], hepatorenal syndrome [89,90,91], and spontaneous bacterial peritonitis [92,93]. However, the role of HA for hyponatremia has not been separately explored in such complications. Another limitation is that the drugs/interventions employed in control groups are heterogeneous among included studies. Furthermore, cardiac disease, hepatorenal syndrome, and infection could also influence the development of hyponatremia in cirrhotic patients [94], but such relevant variables could not be extracted, thereby compromising further subgroup analyses. Finally, the impact of the prevention and correction of hyponatremia by HA on the survival of patients with liver cirrhosis could not be explored in the current meta-analysis due to the absence of relevant data.

In conclusion, HA may be beneficial for the prevention and treatment of hyponatremia in liver cirrhosis. However, its optimal dosage and duration remain unclear and may depend on the patient’s characteristics and response to treatment (e.g., guided by a change of serum albumin and/or sodium level). In the future, the role of HA in the prevention and treatment of hyponatremia in liver cirrhosis with ascites and other complications should be further explored by large-scale well-designed studies, preferably RCTs.

## Figures and Tables

**Table 1:** Characteristics of included studies regarding the prevention of hyponatremia.

First Author (Year)	Country	Study Design	Sample Size (n)	Alcoholic Cirrhosis (%)	Definition of Hyponatremia	Control Group	HA Dose
Ginès (1988) [36]	Spain	RCT	105	65.71% (69/105)	Decrease in serum Na > 5 mmol/L or serum Na < 130 mmol/L after treatment.	No intervention	40 g per time of LVP.
Smart (1990) [64]	UK	RCT	40	45.00% (18/40)	Serum Na < 130 mmol/L.	Filtration	40 g per time of LVP.
Planas (1990) [37]	Spain	RCT	88	67.05% (59/88)	Decrease in serum Na > 5 mmol/L or serum Na < 130 mmol/L after treatment.	Dextran	8 g/L of ascites removed.
Salerno (1991) [38]	Italy	RCT	54	46.30% (25/54)	Decrease in serum Na > 5 mmol/L or serum Na < 130 mmol/L after treatment.	Hemaccel	6 g/L of ascites removed.
Fassio (1992) [39]	Argentina	RCT	41	82.93% (34/41)	Decrease in serum Na > 5 mmol/L or serum Na < 130 mmol/L after treatment.	Dextran	6 g/L of ascites removed.
Garcia-Compean (1993) [40]	Mexico	RCT	35	71.43% (25/35)	Decrease in serum Na > 5 mmol/L or serum Na < 130 mmol/L after treatment.	No intervention	5 g/L of ascites removed.
Hernández Pérez (1995) [41]	Mexico	RCT	16	NA	NA	Dextran	6 g/L of ascites removed.
Ginès (1996) [42]	Spain	RCT	190	70.00% (133/190)	Decrease in serum Na > 5 mmol/L or serum Na < 130 mmol/L after treatment.	Dextran	8 g/L of ascites removed.
Altman (1998) [43]	France	RCT	60	83.33% (50/60)	Decrease in serum Na > 10 mmol/L to serum Na < 120 mmol/L after treatment.	Hydroxyethyl starch	8 g/L of ascites removed.
Gentilini (1999) [44]	Italy	RCT	68	23.53% (16/68)	NA	No intervention	12.5 g/day.
Zaak (2001) [45]	Germany	Cohort	35	88.57% (31/35)	NA	Filtration	5 g/L of ascites removed.
García-Compean (2002) [46]	Mexico	RCT	96	80.21% (77/96)	Decrease in serum Na > 5 mmol/L after treatment.	Dextran	NA
Moreau (2002) [47]	France	RCT	20	85.00% (17/20)	Decrease in serum Na > 5 mmol/L or serum Na < 130 mmol/L after treatment.	Terlipressin	8 g/L of ascites removed.
Sola-Vera (2003) [48]	Spain	RCT	72	55.56% (40/72)	Decrease in serum Na > 10 mmol/L to serum Na < 125 mmol/L after treatment.	Saline	8 g/L of ascites removed.
Moreau (2006) [49]	France	RCT	68	100.00% (68/68)	Decrease in serum Na > 5 mmol/L to serum Na < 130 mmol/L after treatment.	Polygeline	NA
Singh (2006) [50]	India	RCT	40	70.00% (28/40)	Decrease in serum Na > 5 mmol/L to serum Na < 130 mmol/L after treatment.	Terlipressin	8 g/L of ascites removed.
Appenrodt (2008) [52]	Germany	RCT	24	79.20% (19/24)	NA	Midodrine	8 g/L of ascites removed.
Singh (2008) [53]	India	RCT	40	65.00% (26/40)	Decrease in serum Na > 5 mmol/L to serum Na < 130 mmol/L after treatment.	Midodrine	8 g/L of ascites removed.
Abdel-Khalek (2010) [54]	Egypt	RCT	135	NA	Decrease in serum Na > 5 mmol/L to serum Na < 130 mmol/L after treatment.	Hydroxyethyl starch	8 g/L of ascites removed.
Bari (2012) [55]	USA	RCT	25	52.00% (13/25)	NA	Octreotide	8 g/L of ascites removed.
Hamdy (2014) [56]	Egypt	RCT	50	NA	NA	Midodrine	8 g/L of ascites removed.
Khan (2015) [57]	Pakistan	RCT	50	NA	NA	Hemaccel	6 g/L of ascites removed.
Abootalebi (2017) [58]	Iran	RCT	72	NA	NA	Hydroxyethyl starch	5 g/L of ascites removed.
Solà (2018) [61]	Spain	RCT	173	56.07% (97/173)	NA	Placebo	40 g/15 days.
Yosry (2019) [62]	Egypt	RCT	50	0 (0/50)	Decrease in serum Na > 5 mmol/L or serum Na < 130 mmol/L after treatment.	Midodrine	8 g/L of ascites removed.

Abbreviations: HA, human albumin; Na, sodium; LVP, large volume paracentesis; RCT, randomized control trial; NA, not available.

## Data Availability

Data sharing is not applicable to this article as no new data were created in this study.

## Supplementary Materials

The following supporting information can be downloaded at: https://www.mdpi.com/article/10.3390/jcm11195928/s1, Table S1: Characteristics of included studies regarding the treatment of hyponatremia, Table S2: Quality of included cohort studies, Table S3: Meta-regression analysis regarding the human albumin infusion for the prevention of the decreasing of serum sodium level, Table S4: Subgroup analysis of human albumin infusion to prevent the decreasing of serum sodium level in liver cirrhosis, Table S5: Quality of evidence, Figure S1: Risk of bias of RCTs, Figure S2: Publication bias among studies regarding the effect of human albumin infusion on the development of hyponatremia (A) and serum sodium level (B) in liver cirrhosis without hyponatremia, Figure S3: Sensitivity analysis regarding effect of human albumin infusion on serum sodium level in liver cirrhosis without hyponatremia [95].
