# Supplementary material for: Use of Human Albumin Administration for the Prevention and Treatment of Hyponatremia in Patients with Liver Cirrhosis: A Systematic Review and Meta-Analysis
Source: J Clin Med. 2022 Oct 8;11(19):5928. doi: 10.3390/jcm11195928 (PMC9572637; doi:10.3390/jcm11195928)
Supplement: Supplementary file 1 [file jcm-11-05928-s001.zip › jcm-1879034-supplementary.pdf]

Supplementary Table S1. Characteristics of included studies regarding the treatment of hyponatremia.

First author (year)	Country	Sample size (n)	Study design	Population	Control group	Albumin dose
Jalan (2007) Conference Abstract	UK	24	RCT	Cirrhosis with refractory ascites and hyponatremia	Placebo	40g/day
Shen (2017)	USA	146	Cohort	Cirrhosis with hyponatremia	Crystalloid	NA
Bajaj (2018)	USA	1126	Cohort	Cirrhosis with hyponatremia	No intervention	Mean: 225g
China (2021)	UK	206	Cohort (post hoc analysis of an RCT)	Cirrhosis with hyponatremia	Standard care	Mean: 239.4g
Zaccherini (2022)	Italy	149	Cohort (post hoc analysis of an RCT)	Cirrhosis with hyponatremia	Standard care	40g twice weekly for 2weeks, and then 40g weekly

Abbreviations: RCT, randomized control trials; NA, not available.

Supplementary Table S2. Quality of included cohort studies.

Study	Selection				Comparability	Outcome			Total
	Representativeness of the exposed cohort	Selection of the non-exposed cohort	Ascertainment of exposure	Demonstration that outcome of interest was not present at start of study	Comparability of cohorts on the basis of the design or analysis	Assessment of outcome	Was follow-up long enough for outcomes to occur	Adequacy of follow-up of cohorts	
Zaak (2001)	*	*	*	*	**	*	*	\	8
Shen (2017)	*	*	*	\	*	\	*	*	6
Bajaj (2018)	\	\	*	*	**	*	*	*	7
China (2021)	\	\	*	*	**	*	*	*	7
Zaccherini (2022)	\	\	*	*	**	*	*	*	7

Supplementary Table S3. Meta regression analysis regarding the human albumin infusion for the prevention of the decreasing of serum sodium level.

r	Coefficient	Standard error.	t	P	95% Confidence interval	
Region	-0.1431236	0.1678305	-0.85	0.409	-0.5056994	0.2194521
Publication year	0.6619649	0.4044404	1.64	0.126	-0.2117756	1.535705
Sample size	-0.0825302	0.4017505	-0.21	0.840	-0.9504594	0.785399
Type of control group	0.0848842	0.0650131	1.31	0.214	-0.0555681	0.2253364
Target population	-1.623975	0.7065038	-2.30	***0.039***	-3.150283	-0.0976662

Supplementary Table S4. Subgroup analysis of human albumin infusion for prevent the decreasing of serum sodium level in liver cirrhosis.

Subgroups	P value (Effect Size)	Heterogeneity	
		I^2	P value
**Human albumin vs. No intervention**			
OR=1.59, 95%CI=0.99-2.19	<0.00001	81%	0.005
**Human albumin vs. Saline**			
OR=2.30, 95%CI=-0.49-5.09	0.11	\	\
**Human albumin vs. Dextran**			
OR=1.01, 95%CI=0.61-1.41	<0.00001	***0%***	***0.97***
**Human albumin vs. Hydroxyethyl starch**			
OR=0.48, 95%CI=-0.57-1.53	0.37	56%	0.11
**Human albumin vs. Hemaccel**			
OR=0.43, 95%CI=-2.51-3.46	0.78	93%	0.0001
**Human albumin vs. Terlipressin**			
OR=1.70, 95%CI=-1.17-4.57	0.25	\	\
**Human albumin vs. Midodrine**			
OR=0.29, 95%CI=-1.19-1.77	0.70	***33%***	***0.21***
**Human albumin vs. Octreotide**			
OR=0.00, 95%CI=-3.78-3.78	1	\	\

Supplementary Table S5. Quality of Evidence.

Certainty assessment							№ of patients		Effect		Certainty
№ of studies	Study design	Risk of bias	Inconsistency	Indirectness	Imprecision	Other considerations	HSA	Control	Relative (95% CI)	Absolute (95% CI)	
**Role of the HSA infusion for the incidence of hyponatremia (assessed with: OR)**											
18	randomised trials	serious[a]	serious[a]	not serious	not serious	none	55/664 (8.3%)	96/654 (14.7%)	**OR 0.55** (0.38 to 0.80)	**6 fewer per 100** (from 9 fewer to 3 fewer)	⨁⨁◯◯ Low
**Role of the HSA infusion for the decreasing of serum sodium level (assessed with: MD)**											
19	randomised trials	serious[a,b]	serious[a]	not serious	not serious	none	654	641	-	MD **0.95 higher** (0.47 higher to 1.43 higher)	⨁⨁◯◯ Low
**Role of the HSA infusion for the treatment of hyponatremia (assessed with: OR)**											
2	observational studies	very serious[c]	very serious[b]	not serious	not serious	none	570/848 (67.2%)	234/422 (55.5%)	**OR 1.50** (1.17 to 1.92)	**97 more per 1,000** (from 38 more to 150 more)	⨁◯◯◯ Very low

CI: confidence interval; **MD:** mean difference; **OR:** odds ratio

Explanations

a. The control group in these studies are different.
b. There is heterogeneity among the included patients.
c. No clear definition of hyponatremia.

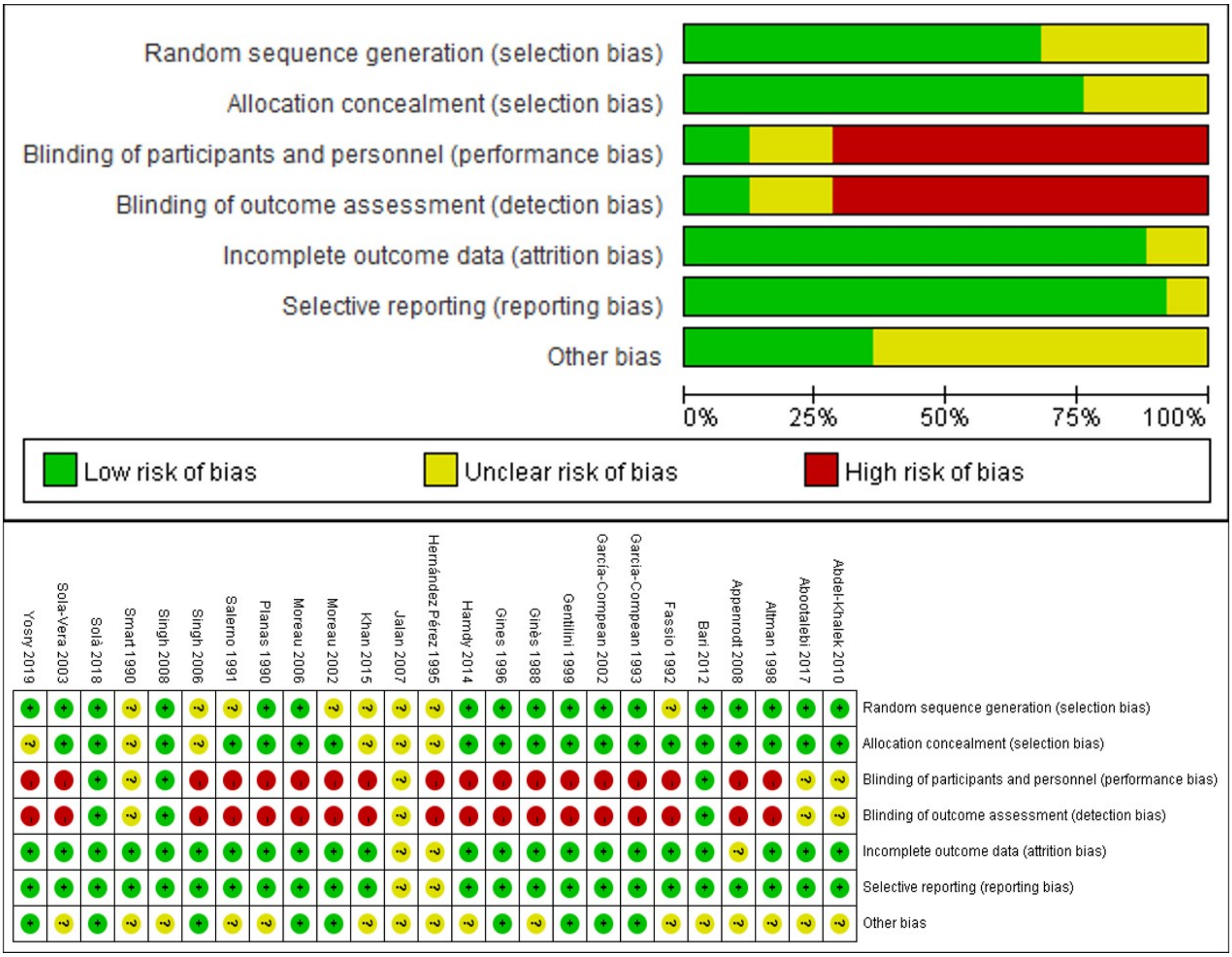

Supplementary Figure S1. Risk of bias of RCTs.

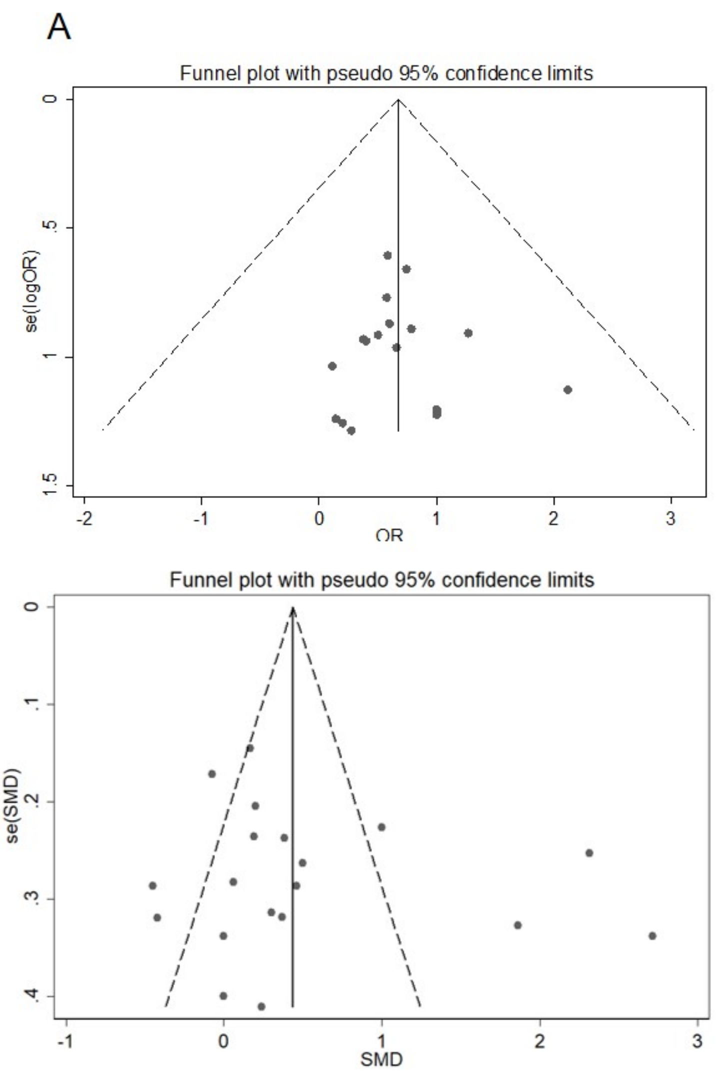

Supplementary Figure S2. Publication bias among studies regarding effect of human albumin infusion on development of hyponatremia (A) and serum sodium level (B) in liver cirrhosis without hyponatremia.

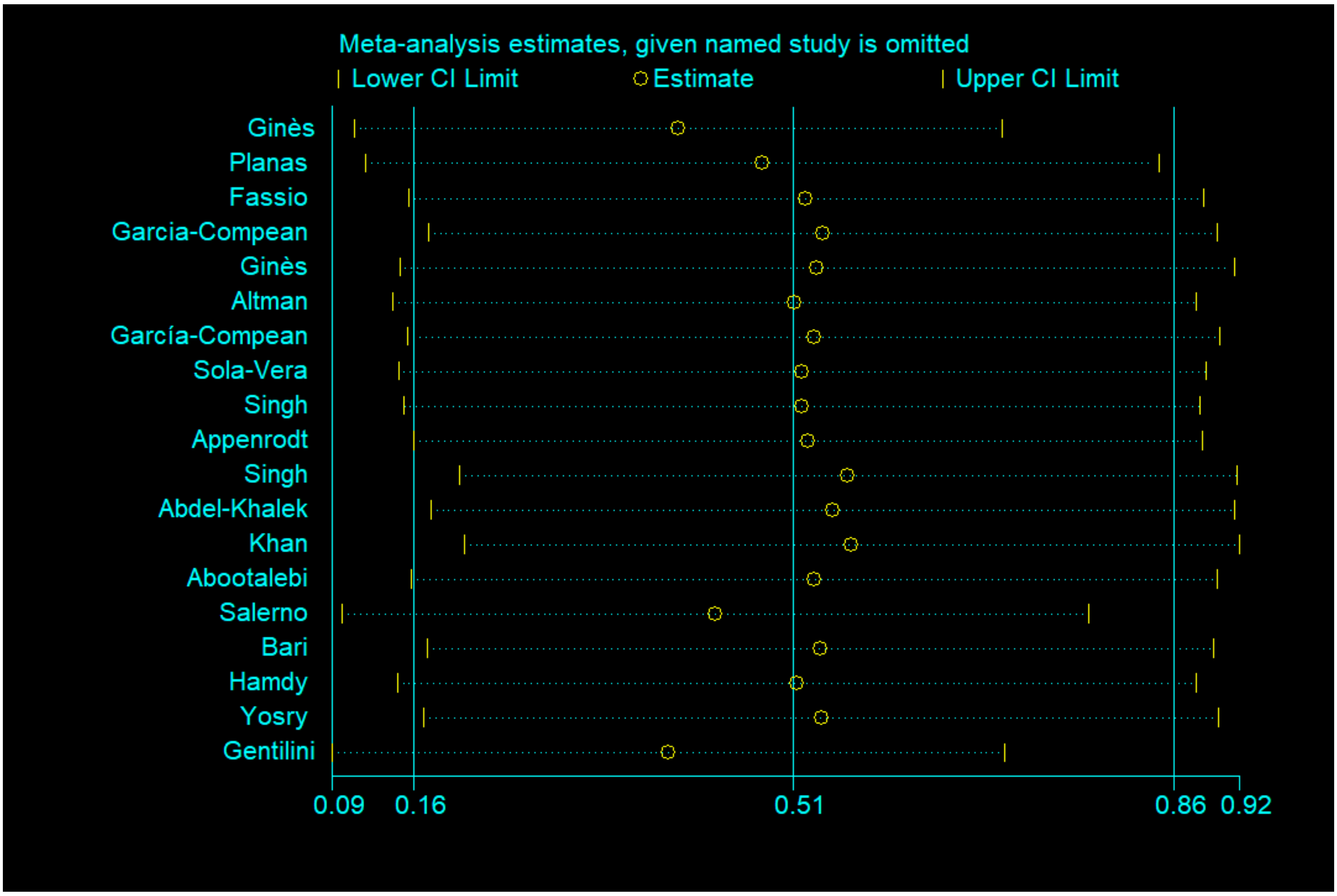

Supplementary Figure S3. Sensitivity analysis regarding effect of human albumin infusion on serum sodium level in liver cirrhosis without hyponatremia.

PRISMA 2009 checklist

Section/topic	#	Checklist item	Reported on page #
**TITLE**			
Title	1	Identify the report as a systematic review, meta-analysis, or both.	P1
**ABSTRACT**			
Structured summary	2	Provide a structured summary including, as applicable: background; objectives; data sources; study eligibility criteria, participants, and interventions; study appraisal and synthesis methods; results; limitations; conclusions and implications of key findings; systematic review registration number.	P2
**INTRODUCTION**			
Rationale	3	Describe the rationale for the review in the context of what is already known.	P3
Objectives	4	Provide an explicit statement of questions being addressed with reference to participants, interventions, comparisons, outcomes, and study design (PICOS).	P3
**METHODS**			
Protocol and registration	5	Indicate if a review protocol exists, if and where it can be accessed (e.g., Web address), and, if available, provide registration information including registration number.	P4
Eligibility criteria	6	Specify study characteristics (e.g., PICOS, length of follow-up) and report characteristics (e.g., years considered, language, publication status) used as criteria for eligibility, giving rationale.	P4
Information sources	7	Describe all information sources (e.g., databases with dates of coverage, contact with study authors to identify additional studies) in the search and date last searched.	P4
Search	8	Present full electronic search strategy for at least one database, including any limits used, such that it could be repeated.	P4

Study selection	9	State the process for selecting studies (i.e., screening, eligibility, included in systematic review, and, if applicable, included in the meta-analysis).	P4
Data collection process	10	Describe method of data extraction from reports (e.g., piloted forms, independently, in duplicate) and any processes for obtaining and confirming data from investigators.	P4
Data items	11	List and define all variables for which data were sought (e.g., PICOS, funding sources) and any assumptions and simplifications made.	P4
Risk of bias in individual studies	12	Describe methods used for assessing risk of bias of individual studies (including specification of whether this was done at the study or outcome level), and how this information is to be used in any data synthesis.	P4
Summary measures	13	State the principal summary measures (e.g., risk ratio, difference in means).	P4
Synthesis of results	14	Describe the methods of handling data and combining results of studies, if done, including measures of consistency (e.g., I^2) for each meta-analysis.	P4-P5

**Section/topic**	**#**	**Checklist item**	**Reported on page #**
Risk of bias across studies	15	Specify any assessment of risk of bias that may affect the cumulative evidence (e.g., publication bias, selective reporting within studies).	P4
Additional analyses	16	Describe methods of additional analyses (e.g., sensitivity or subgroup analyses, meta-regression), if done, indicating which were pre-specified.	P4-P5
**RESULTS**			
Study selection	17	Give numbers of studies screened, assessed for eligibility, and included in the review, with reasons for exclusions at each stage, ideally with a flow diagram.	P6

Study characteristics	18	For each study, present characteristics for which data were extracted (e.g., study size, PICOS, follow-up period) and provide the citations.	P6
Risk of bias within studies	19	Present data on risk of bias of each study and, if available, any outcome level assessment (see item 12).	P6
Results of individual studies	20	For all outcomes considered (benefits or harms), present, for each study: (a) simple summary data for each intervention group (b) effect estimates and confidence intervals, ideally with a forest plot.	P6
Synthesis of results	21	Present results of each meta-analysis done, including confidence intervals and measures of consistency.	P6-P8
Risk of bias across studies	22	Present results of any assessment of risk of bias across studies (see Item 15).	P6-P8
Additional analysis	23	Give results of additional analyses, if done (e.g., sensitivity or subgroup analyses, meta-regression [see Item 16]).	P6-P8
**DISCUSSION**			
Summary of evidence	24	Summarize the main findings including the strength of evidence for each main outcome; consider their relevance to key groups (e.g., healthcare providers, users, and policy makers).	P9-P10
Limitations	25	Discuss limitations at study and outcome level (e.g., risk of bias), and at review-level (e.g., incomplete retrieval of identified research, reporting bias).	P10
Conclusions	26	Provide a general interpretation of the results in the context of other evidence, and implications for future research.	P10
**FUNDING**			
Funding	27	Describe sources of funding for the systematic review and other support (e.g., supply of data); role of funders for the systematic review.	P1

From: Moher D, Liberati A, Tetzlaff J, Altman DG, The PRISMA Group (2009). Preferred Reporting Items for Systematic Reviews and Meta-Analyses: The PRISMA Statement. PLoS Med 6(7): e1000097. doi:10.1371/journal.pmed1000097

For more information, visit: **www.prisma-statement.org**.
